# Pharmacokinetic and metabolomic analyses of Mangiferin calcium salt in rat models of type 2 diabetes and non-alcoholic fatty liver disease

**DOI:** 10.1186/s40360-020-00438-x

**Published:** 2020-08-06

**Authors:** He Lin, Houlei Teng, Wei Wu, Yong Li, Guangfu Lv, Xiaowei Huang, Wenhao Yan, Zhe Lin

**Affiliations:** 1 College of Pharmacy, Changchun University of Chinese Medicine, Changchun, China; 2 Changzhou Deze Drug Research Co., Ltd, Changzhou, China

**Keywords:** Mangiferin calcium salt, Diabetes, NAFLD, Pharmacokinetics, Metabolomics, Bioavailability

## Abstract

**Background:**

Non-alcoholic fatty liver is one of the most common comorbidities of diabetes. It can cause disturbance of glucose and lipid metabolism in the body, gradually develop into liver fibrosis, and even cause liver cirrhosis. Mangiferin has a variety of pharmacological activities, especially for the improvement of glycolipid metabolism and liver injury. However, its poor oral absorption and low bioavailability limit its further clinical development and application. The modification of mangiferin derivatives is the current research hotspot to solve this problem.

**Methods:**

The plasma pharmacokinetic of mangiferin calcium salt (MCS) and mangiferin were monitored by HPLC. The urine metabolomics of MCS were conducted by UPLC-Q-TOF-MS.

**Results:**

The pharmacokinetic parameters of MCS have been varied, and the oral absorption effect of MCS was better than mangiferin. Also MCS had a good therapeutic effect on type 2 diabetes and NAFLD rats by regulating glucose and lipid metabolism. Sixteen potential biomarkers had been identified based on metabolomics which were related to the corresponding pathways including Pantothenate and CoA biosynthesis, fatty acid biosynthesis, citric acid cycle, arginine biosynthesis, tryptophan metabolism, etc.

**Conclusions:**

The present study validated the favorable pharmacokinetic profiles of MCS and the biochemical mechanisms of MCS in treating type 2 diabetes and NAFLD.

## Background

Diabetes is one of the most common chronic metabolic diseases, and its incidence is gradually increasing. According to the International Diabetes Federation, the 463 million people with diabetes worldwide account for about 9.3% of the global population in 2019, of which 80% come from low- and middle-income countries. It is estimated that 700 million people will account for 10.9% of the world population by 2045 [1]. Non-alcoholic fatty liver (NAFLD) is a metabolic stress liver injury, including non-alcoholic simple fatty liver, non-alcoholic steatohepatitis and related cirrhosis [2, 3]. NAFLD is currently the most common liver disease in the world and the common comorbidities of diabetes. It accounts for about 75% of patients with type 2 diabetes [4, 5]. It can cause further disorders of glucose and lipid metabolism, and gradually progress to liver fibrosis, and even cause Cirrhosis [6]. The coexistence of two diseases could affect the health of patients seriously [7]. Insulin resistance (IR) is currently recognized as one of the main risk factors for non-alcoholic fatty liver. It refers to the reduced sensitivity of the body to insulin, the inability to effectively synthesize and metabolize glucose. Then excessive insulin is compensatively secreted into the blood, causing hyperinsulinemia [8, 9]. Meanwhile IR prevents insulin from efficiently inhibiting lipase activity. The increase of lipase activity will cause a large amount of adipose tissue to be broken down, and excess free fatty acids will enter the liver through the hepatic portal vein, causing fatty liver [10, 11]. IR can also trigger oxidative stress, inflammation that promotes the deterioration of NAFLD, causing inflammation infiltration, necrosis, and even fibrosis in the liver [12, 13].

Mangiferin (2-beta-D-glucopyranosyl-1,3,6,7-tetrahydroxyxanthone, MGN) is a natural C-glucoside xanthone, which is predominantly in the fruits, leaves, and bark of *Mangifera indica* L. and some other medical plants including Anemarrhena asphodeloides Bge., *Belamcanda chinensis* (L.) DC etc. [14, 15]. It has shown many kinds of biological activities and pharmacological actions such as antioxidative, antidiabetic, hypolipidemic, antiviral, immunomodulatory, anticancer, analgesic and hepatoprotective effects [16–20]. But the characteristic of its low aqueous solubility and low fat solubility can affect the absorption process of drugs in vivo, which leads to a low bioavailability [21, 22]. It makes us have to suffer such problems like mangiferin is hard to further develop a new medicine and its clinical application has certain limitation.

**Fig. 1:**
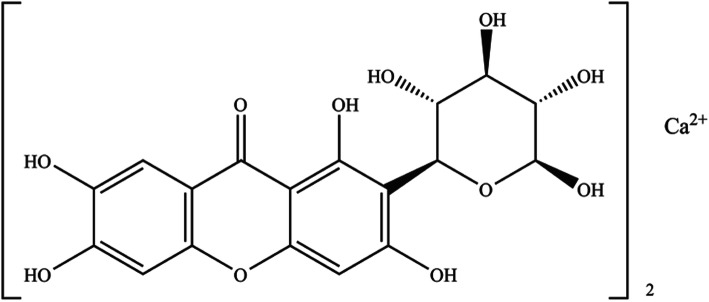
Chemical structure of mangiferin calcium salt

Mangiferin calcium salt (MCS) is a new salt of mangiferin which proposed to be an insulin sensitizer (Fig. 1) [23, 24]. In the present study, the pharmacokinetic profiles of MCS in rats were evaluated to clarify the impact of single and repeated administration on its main pharmacokinetic parameters. A comparison between the major pharmacokinetic between MCS and mangiferin was subsequently executed. Metabolomics was performed with rats urine samples collected from oral administration of MCS. As our knowledge, this is the first integrated study of pharmacokinetics and metabolomics on MCS. The results of this assessment will contribute to further development of MCS as pharmaceutical products and explore the underlying mechanism of MCS in the treatment of type 2 diabetes and NAFLD.

## Methods

### Chemicals and materials

Mangiferin calcium salt (MCS, yellow green powder, purity: 95.25%), Mangiferin (yellowish powder, purity: 98%) was provided by Changzhou Deze Pharmaceutical Research Co. Ltd. (Changzhou, China). Mangiferin (purity: 98.1%), rutin (purity: 91.9%) as reference substance were obtained from the National Institute for the Control of Pharmaceutical and Biological Products (Beijing, China). Heparin sodium was obtained from Shanghai Huishi biochemical reagent Co., Ltd. (Shanghai, China). Acetonitrile Methanol and formic acid (HPLC grade) were obtained from Tedia Company, Inc. (Ohio, USA). Ultrapure water was produced using a Milli-Q plus (Milford, MA, USA) water purification system. Leucine enkephalin was obtained from Waters (Milford, USA). Xanthurenic acid, 5-L-Glutamyl-taurine, Citric acid, Pantothenic acid, Uric acid, Riboflavin and 3-Hydroxyanthranilic acid were obtained from Sigma-Aldrich (St. Louis, MO, USA).

### Animals

Sprague-Dawley rats (male and female, weighting 200-230 g) were obtained from Changchun Yisi Laboratory Animal Technology Co., Ltd. (Changchun, China). Rats were housed with free access to food and water under standard conditions (temperature 20–24 °C, humidity 40–60%, 12-h light/dark cycle). All experimental animals were finally euthanized by CO_2_ inhalation. The study complied with the guidelines of the research commitment institution and its administrative region, as the Jilin Province Experimental Animal Management Ordinance and Changchun University of Chinese Medicine Laboratory Animal Management Measures. All experiments were approved by the Laboratory Animals Ethics Committee, Changchun University of Chinese Medicine.

### Administration and plasma samples collection

MCS and mangiferin were given by gavages according to 60 mg/kg, 240 mg/kg, 960 mg/kg doses as single administration. Rats were fasted 12 h before the experiment and water was taken freely. In the experiment day the administration was according to the predetermined dose. Serial blood samples were collected from the orbital venous plexus (0.3–0.5 mL) at 0 h, 0.5 h, 1.0 h, 1.5 h, 2.0 h, 3.0 h, 4.0 h, 6.0 h, 10.0 h, 12.0 h, 24.0 h after administration.

MCS and mangiferin were given by gavages according to 240 mg/kg dose, once a day for 7 days as multiple administrations. Blood samples were collected from the orbital venous plexus (0.3–0.5 mL) on 1, 2, 3, 4, 5, 6 days before dosing. For the last administration, Serial blood samples were collected at 0 h, 0.5 h, 1.0 h, 1.5 h, 2.0 h, 3.0 h, 4.0 h, 6.0 h, 10.0 h, 12.0 h, 24.0 h.

The blood samples placed in a centrifuge tube with heparin, 10,000 rpm centrifuge 10 min. After the centrifugation, reserve the plasma in − 20 °C refrigerator.

### Pharmacokinetic analysis

The 200 μL of plasma sample was placed in a 1.5 mL centrifuge tube, added internal standard solution (10 μg/mL rutin standard solution) 25 μL, (0 h plasma used methanol 25 μL to instead), methanol 25 μL (added mangiferin standard solution 25 μL), added 0.9 mL Acetonitrile-acetic acid (9: 1), swirl mixed 3 min, 6000 rpm centrifuged 10 min, supernatant was dried in vacuum at 50 °C, added mobile phase 100 μL to the residue, swirl mixed 2 min, 6000 rpm centrifuged 10 min, the supernatant was injected into High performance liquid chromatography (HPLC). Chromatographic separations were achieved using a Discovery C18 column (250*4.60 mm I.D, 5 μm, Supelco Company, USA). The mobile phase used for the separation consisted of Acetonitrile and 0.10% phosphoric acid (25:75, v/v) delivered at 1 ml/min flow rate. The detection wavelength was set at 318 nm and all measurements were performed at 30 °C.

The pharmacokinetic parameters were calculated using DAS software, and select the weighting factors to fit the atrioventricular model.

### Type 2 diabetes and NAFLD model construction and administration

The SD rats were fed high-fat feed (recipe: 12% lard, 0.5% cholate, 1% cholesterol, 5% sucrose, 81.5% basic nutritional feed). At the end of the 12th week, streptozotocin (STZ) (30 mg/kg) was intraperitoneally injected into rats to induce type 2 diabetes complicated with NAFLD model. The rats were randomly divided into the following four groups: Blank control group (BG, *n* = 7), model control group (MG, n = 7) were administered with distilled water intragastrically. MCS High-dose group (MHG, n = 7), Medium dose group (MMG, n = 7), Low-dose group (MLG, n = 7) were administered intragastrically with MCS at doses of 480 mg/kg, 240 mg/kg, 120 mg/kg.

### Pharmacodynamics

Blood was collected and centrifuged at 4500 rpm low temperature centrifuge for 15 min to separate serum. Detect the fasting blood glucose (FBG), fasting insulin (FINS), triglyceride (TG), total cholesterol (TC), aspartate aminotransferase (AST), alanine aminotransferase (ALT) and gamma-glutamyl transpeptadase (GGT) content in rat serum. The rat liver was taken stained with hematoxylin and eosin (H&E).

### Metabolomics analysis

Urine samples were collected and centrifuged at 10,000 rpm for 10 min, filtered through a 0.22 μm filter membrane. Supernatant was transferred to fresh vials for ultra-performance liquid chromatography coupled with quadrupole time-of-flight mass spectrometry (UPLC-Q-TOF-MS) analysis. For metabolomics analysis, the samples (each 5 μL) were injected onto a Waters ACQUITY UPLC BEH C18 Column (1.7 m, 2.1 mm × 50 mm) kept at 30 °C and at a flow rate of 0.4 mL/min using a Waters ACQUITY UPLC system coupled with a Q-TOF SYNAPT G2 High Definition Mass Spectrometer (Waters, USA). Acetonitrile (A) and 0.1% aqueous formic acid (v/v) (B) were used as gradient mobile phase. The gradient elution of A was performed as follows: 5–30% A at 0–6 min, 30–60% A at 6–10 min, 60–100% A at 10–12 min, 100–5% A at 12–12.1 min and then kept at 5% A for 3 min. The positive and negative ion (ESI) modes were used in MS analysis. The source temperature was set to 120 °C. The desolvation gas temperature was set to 400 °C and the flow was set to 800 L/h. The capillary, cone and extraction cone voltages were 3.0 kV, 35 V, 5.0 V in positive ion mode and 2.0 kV, 35 V, 5.0 V in negative ion mode. The full-scan mode was from 100 to 1000 Da. Accurate mass was maintained by Leucine enkephalin. MSE was applied for the MS/MS analysis with the high collision energy on 25-35 eV and the low collision energy on 4 eV.

The quality control (QC) samples were used for method validation, which were obtained by mixing 100 μL of each sample. In order to avoid errors during the entire analysis process, the QC samples were run once every 5 samples to measure the stability of the system.

### Data processing and statistical analysis

The sample was detected by UPLC-Q-TOF-MS to obtain the total ion current chromatogram of the sample. The raw data files were processed with MassLynx V4.1 and MarkerLynx Application Manager (Waters, USA) for peak detection, alignment and normalization. Multivariate analysis was performed by principal component analysis (PCA) and orthogonal projection to latent structures squares-discriminant analysis (OPLS-DA) with the EZinfo 2.0 software. All values are expressed as the mean ± SD. An independent sample t-test between groups was used to evaluate the significant difference (*p* < 0.05)using SPSS statistics 13.0 software.

## Results

### Comparison of pharmacokinetic parameters after single administration of MCS and mangiferin

**Fig. 2:**
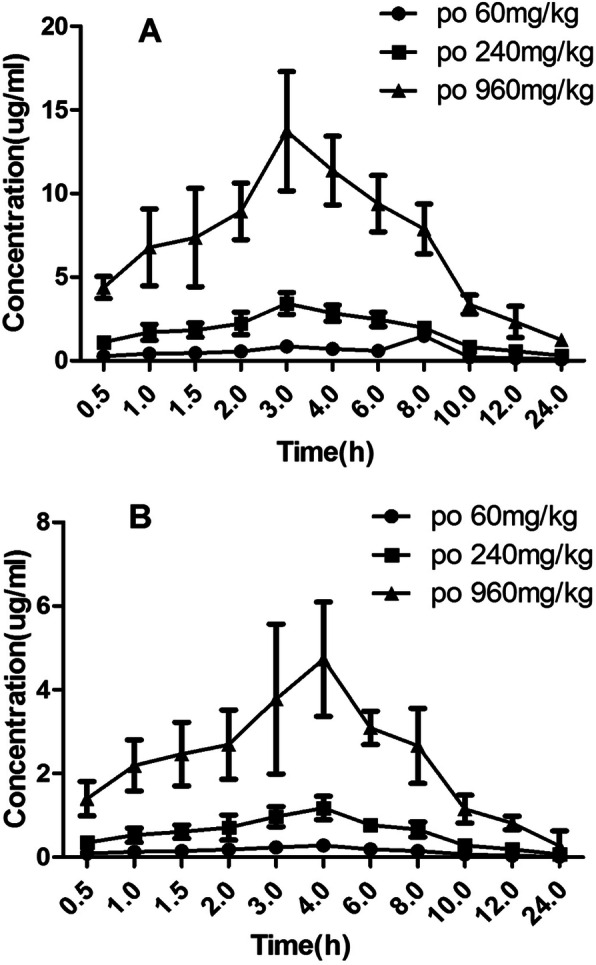
The mean plasma concentration-time curves of MCS and mangiferin in different dosage. **a** MCS, **b** mangiferin

The mean plasma concentration-time curves of MCS and mangiferin in different dosage are showed in Fig. 2. The main pharmacokinetic parameters are summarized in Table 1. As be seen in Table 1, after a single administration of 240 mg/kg, compared with AUC(0-t) (9187.50 μg/L•h), AUC(0-∞) (9723.18 μg/L•h), Tmax (4.02 h), Cmax (1.18 μg/ml) of mangiferin, AUC(0-t) (28,126.50 μg/L•h), AUC(0-∞) (30,981.65 μg/L•h), Cmax (3.42 μg/ml) of MCS are significantly increased (*P* < 0.05), Tmax (2.99 h) is significantly decreased (P < 0.05). MCS has better oral absorption than mangiferin.

**Table 1:** Pharmacokinetic parameters after single administration of Mangiferin calcium salt (MCS) and mangiferin in rats (*n* = 6)

Parameters	Mangiferin calcium salt (MCS) (Mean ± SD)			Mangiferin (Mean ± SD)		
	60 mg/kg	240 mg/kg	960 mg/kg	60 mg/kg	240 mg/kg	960 mg/kg
AUC(0-t)(μg/L·h)	6988.35 ± 1537.44	28,126.50 ± 6750.48*	111,771.00 ± 32,413.59	2200.00 ± 462.89	9187.50 ± 2021.15	37,077.50 ± 11,494.23
AUC(0-∞)(μg/L·h)	7714.49 ± 2005.77	30,981.65 ± 8674.40*	123,314.62 ± 38,227.53	2366.16 ± 567.88	9723.18 ± 2430.80	39,101.95 ± 12,121.60
MRT(0-t)(h)	7.28 ± 1.67	7.27 ± 0.96	7.28 ± 0.98	6.90 ± 1.45	6.89 ± 1.58	6.94 ± 1.87
MRT(0-∞)(h)	9.73 ± 0.98	9.65 ± 0.77	9.71 ± 1.94	8.69 ± 2.17	8.25 ± 2.47	8.21 ± 1.89
T1/2(kα)(h)	1.59 ± 0.13	1.60 ± 0.19	1.57 ± 0.20	1.66 ± 0.22	1.70 ± 0.19	1.72 ± 0.24
T1/2(ke)(h)	3.27 ± 0.52	3.34 ± 0.47	3.36 ± 0.47	3.15 ± 0.41	3.29 ± 0.46	3.30 ± 0.46
Tmax(h)	3.11 ± 0.25	2.99 ± 0.21*	3.06 ± 0.12	4.11 ± 0.33	4.02 ± 0.28	3.97 ± 0.36
Cmax(μg/ml)	0.86 ± 0.21	3.42 ± 0.65*	13.73 ± 3.57	0.28 ± 0.04	1.18 ± 0.28	4.73 ± 1.37
V/F(c) (L/kg)	43.50 ± 8.27	45.11 ± 13.98*	45.54 ± 14.12	134.17 ± 41.59	142.41 ± 41.30	133.34 ± 34.67
CL/F(S)(L/kg·h)	9.23 ± 1.57	9.36 ± 2.53*	9.40 ± 2.54	29.55 ± 7.68	30.05 ± 8.71	28.00 ± 7.12

Compared with mangiferin dosage of 240 mg/kg group, *p < 0.05

### Comparison of pharmacokinetic parameters after multiple administration of MCS and mangiferin

**Fig. 3:**
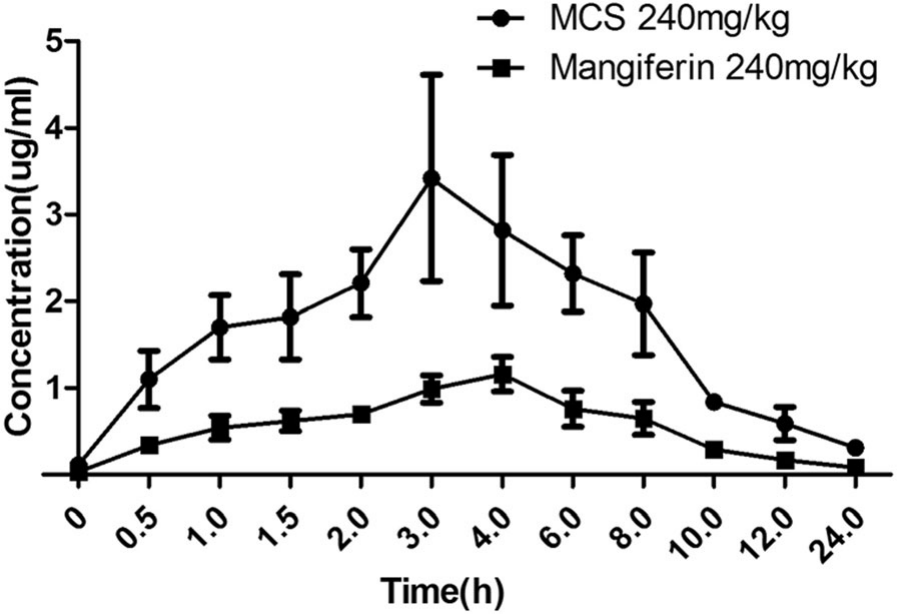
The comparison of mean plasma concentration-time curves of MCS and mangiferin after multiple oral administrations in dosage of 240 mg/kg

The comparison of mean plasma concentration-time curves of MCS and mangiferin after multiple oral administration in dosage of 240 mg/kg are showed in Fig. 3. The main pharmacokinetic parameters are summarized in Table 2. As be seen in Table 2, after a multiple administration of 240 mg/kg, compared with AUC(0-t) (9075.00 μg/L•h), AUC(0-∞) (9729.04 μg/L•h), Tmax (4.05 h), Cmax (1.16 μg/ml) of mangiferin, AUC(0-t) (27,871.50 μg/L•h), AUC(0-∞) (30,789.50 μg/L•h), Cmax (3.42 μg/ml) of MCS are significantly increased (*P* < 0.05), Tmax (3.02 h) is significantly decreased (P < 0.05). In addition, the main pharmacokinetic parameters of multiple and single administration of MCS have no significant difference, indicating that the absorption of MCS in rats is constant basically, and don’t change with continuous administration. MCS almost has no accumulation in the body after multiple doses of administration.

**Table 2:** Pharmacokinetic parameters after multiple administration of Mangiferin calcium salt (MCS) and mangiferin in rats (*n* = 6)

Parameters	Mangiferin calcium salt (MCS)	Mangiferin
	240 mg/kg (Mean ± SD)	
AUC(0-t)(μg/L·h)	27,871.50 ± 9197.60*	9075.00 ± 2631.75
AUC(0-∞)(μg/L·h)	30,789.48 ± 11,084.21*	9729.04 ± 2724.13
MRT(0-t)(h)	7.31 ± 1.82	6.93 ± 0.97
MRT(0-∞)(h)	9.77 ± 2.40	8.63 ± 1.05
T1/2(kα)(h)	0.77 ± 0.08	0.84 ± 0.11
T1/2(ke)(h)	5.54 ± 0.72	4.92 ± 0.63
Tmax(h)	3.02 ± 0.09*	4.05 ± 0.36
Cssmin(μg/ml)	0.12 ± 0.02*	0.03 ± 0.01
Cmax(μg/ml)	3.42 ± 1.19*	1.16 ± 0.20
Cavg(μg/ml)	1.16 ± 0.09*	0.38 ± 0.03
V/F(c) (L/kg)	75.73 ± 18.93*	215.53 ± 40.95
CL/F(S)(L/kg·h)	9.48 ± 1.52*	30.36 ± 8.50
FI(%)	2.84 ± 0.19	2.97 ± 0.21

Compared with mangiferin group, *p < 0.05

### Pharmacodynamics study

**Fig. 4:**
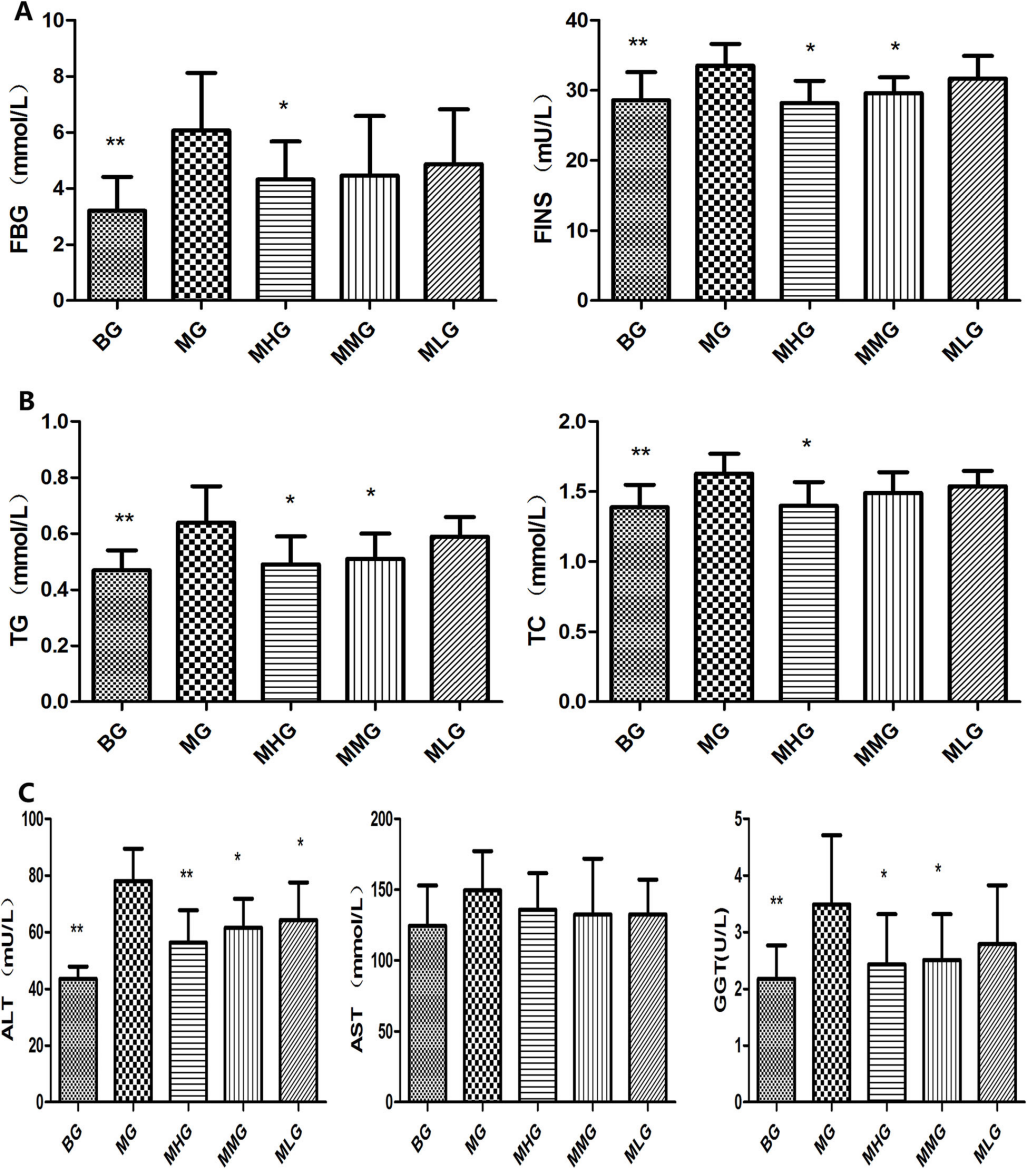
The effect of MCS on type 2 diabetes patients with NAFLD model rat. **a** content of serum FBG and FINS, **b** level of serum TG and TC, **c** activities of serum ALT, AST and GGT

Type 2 diabetes patients with NAFLD often suffered from glucose and lipid metabolism disorder, and present with abnormally high fasting blood glucose, fasting insulin and HOMA-IR [25]. As our previous study (Fig. 4) [26], the serum FBG and FINS content of MG were higher than BG significantly (*P* < 0.01). Compared with MG, the level of serum FBG, FINS in MHG and MMG decreased significantly after treated with MCS (*P* < 0.05). It revealed that MCS could better improve insulin resistance. Dyslipidemia is also one of the important clinical manifestations of type 2 diabetes patients with NAFLD. Compared with BG, significant increase could be observed in serum TG, TC level in MG (*p* < 0.01). After the treatment with MCS, the concretion of serum TG, TC in MHG and MMG decreased significantly (*p* < 0.05). It revealed that MCS could reduce the blood lipid in model rats. ALT, AST, GGT are the most significant diagnostic indicator for patients with NAFLD. Compared with BG, serum ALT and GGT activities of MG increased significantly (*p* < 0.01). After the treatment with MCS, the activities of serum ALT and GGT in MHG and MMG decreased significantly (p < 0.01, p < 0.05). It revealed that MCS could improve abnormal liver function in model rats.

**Fig. 5:**
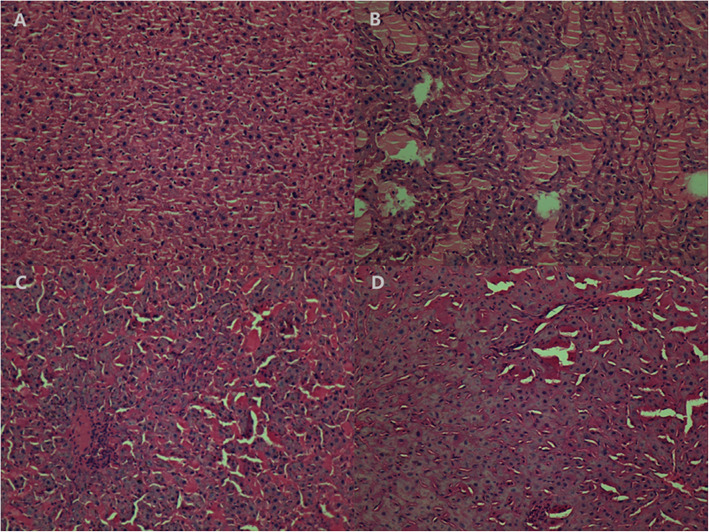
The histological examination of liver tissue (magnification×200). The data are representative H&E stained sections from each group. **a** Blank control group, BG, **b** model control group, MG, **c** MCS High-dose group, MHG, **d** Medium dose group, MMG

Histological analysis showed that livers of the MG rats had lobular structures with blurred boundaries, Irregular cell cords, and hepatic sinusoidal compression became smaller or disappears, liver cells showed diffuse fat-like changes, a large number of inflammatory cells infiltration could be seen in the liver lobule, even several inflammatory necrosis merged with each other (Fig. 5).

### Metabolomics study

**Fig. 6:**
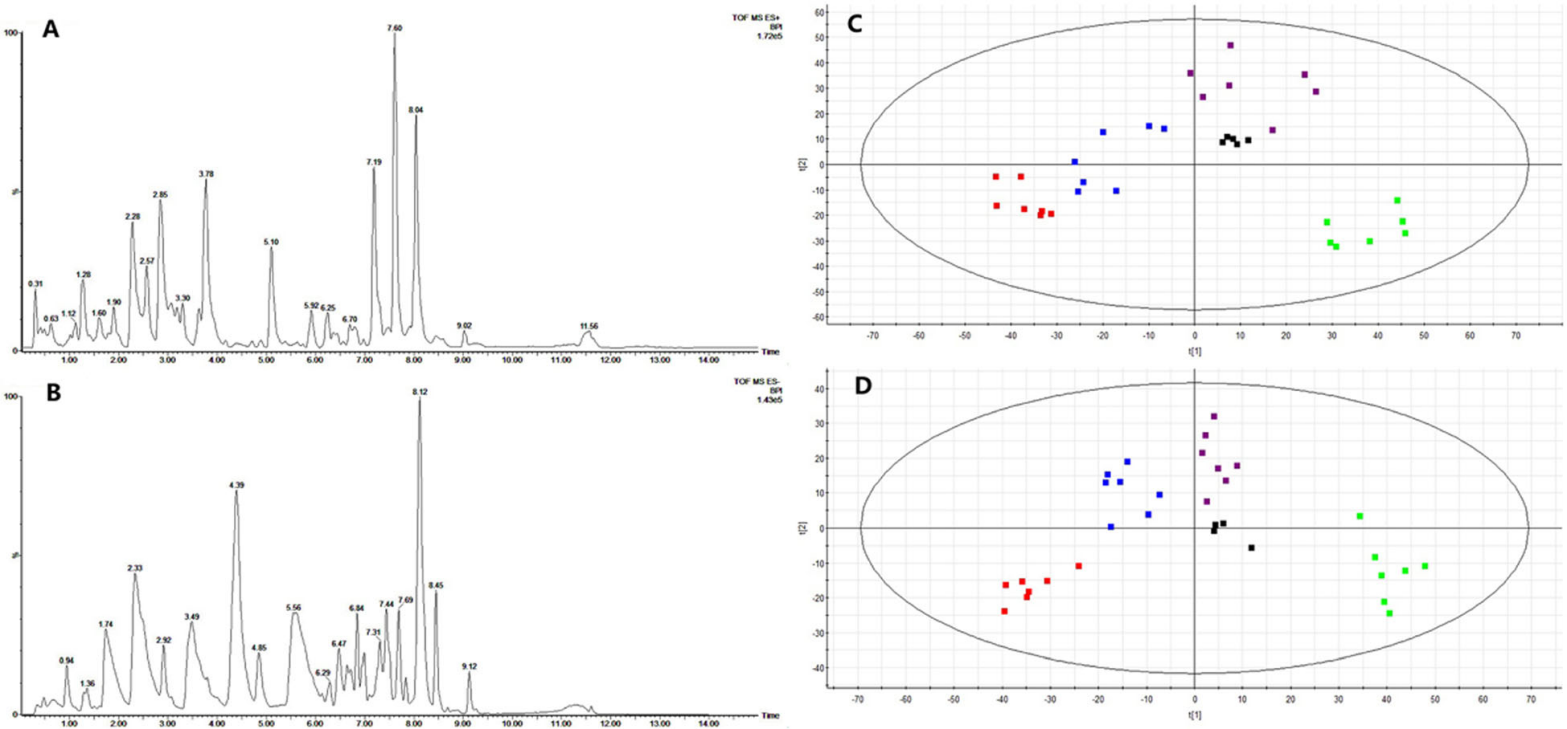
PCA score plots of urine metabolic profiling of BG (red), MG (green), MHG (blue), MMG (violet) and QCs (black) in positive mode (**a**) and negative mode (**b**)

The system of UPLC-Q-TOF-MS is used for urinary sample separation and data collection. Metabolic profiling was acquired in the ESI+ and ESI- modes. The representative based peak intensity (BPI) chromatograms in positive and negative ion modes are showed in Fig. 6a, b. PCA was performed as an unsupervised pattern recognition method to analyze the holistic metabolic variations in different groups and QCs. It can be seen from the PCA score chart (Fig. 6c, d) that the urine samples of four groups can be clearly separated in the positive ion mode (R2X = 0.679, Q2 = 0.410) and the negative ion mode (R2X = 0.596, Q2 = 0.402). The QC samples are relatively compact in both positive ion mode and negative ion mode, revealing that the stability of the analytical system is good. BG and MG are distributed obviously in different regions, indicating that the metabolism of type 2 diabetes with NAFLD model rats has changed. MHG and MMG are close to BG which implies that the metabolic profile of MHG and MMG are returning to normal after administration of MCS.

### Potential biomarkers and metabolic pathway analysis

**Fig. 7:**
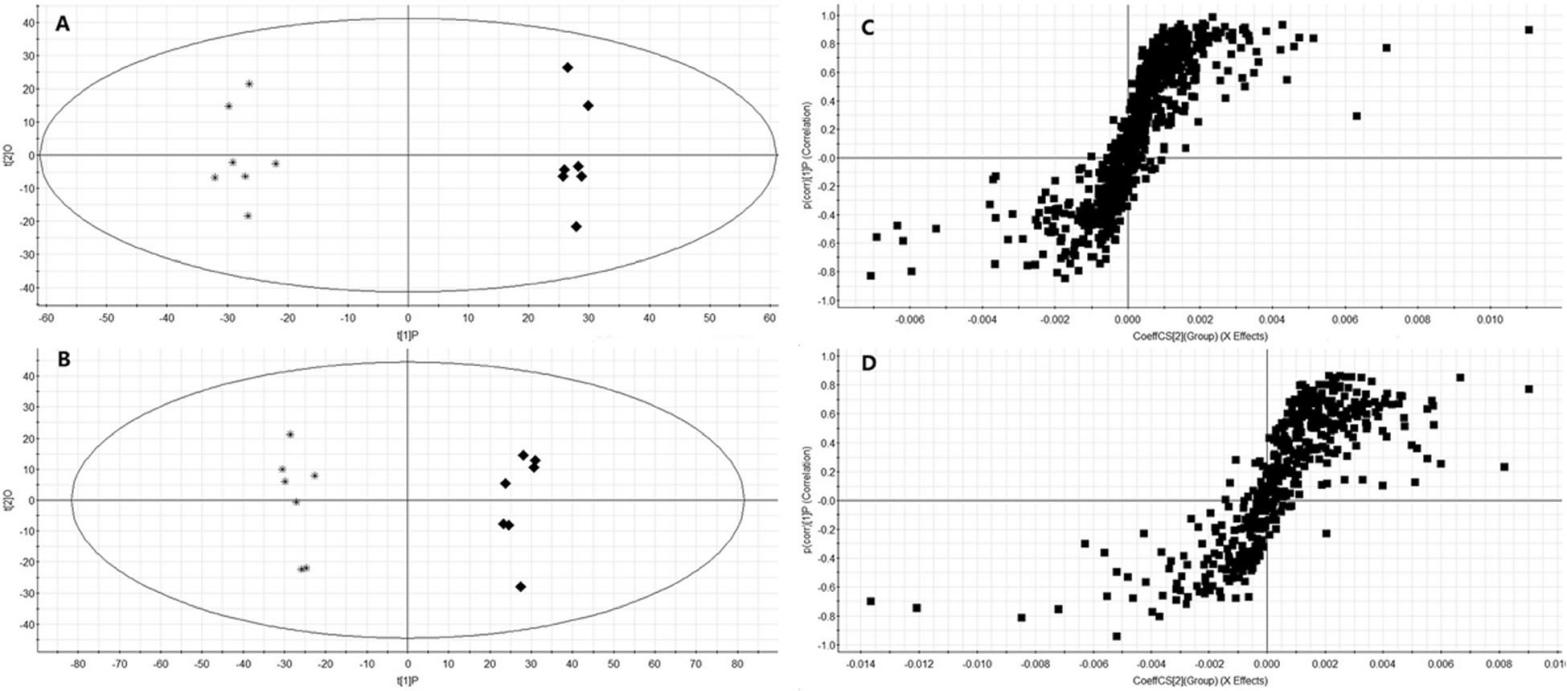
OPLS-DA score plots of urine metabolic profiling of MG (■) and MHG (*) in positive mode (**a**) and negative mode (**b**) and OPLS-DA S-plots in positive mode (**c**) and negative mode (**d**)

**Fig. 8:**
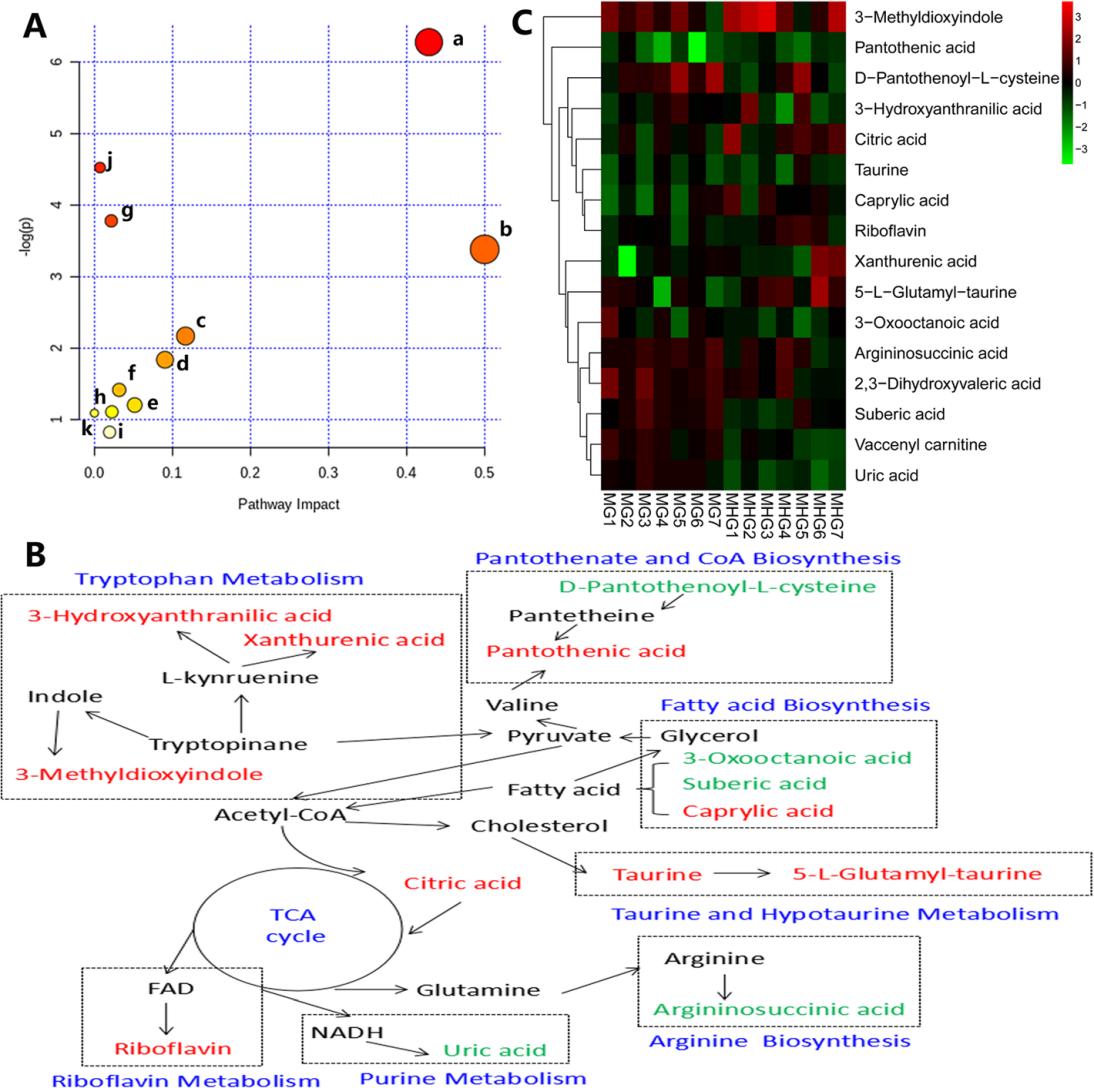
Correlation networks of potential biomarkers and heatmap of metabolites responding to MCS. **a** Metabolic pathway enrichment analysis (from a to k are Taurine and hypotaurine metabolism, Pantothenate and CoA biosynthesis, Alanine, aspartate and glutamate metabolism, Riboflavin metabolism, Arginine biosynthesis, Citrate cycle (TCA cycle), Glyoxylate and dicarboxylate metabolism, Tryptophan metabolism, Primary bile acid biosynthesis, Fatty acid biosynthesis and Purine metabolism), **b** Metabolic pathway networks analysis (the red color indicates up-regulated level; the green color indicates down-regulated level), **c** Heatmap of metabolites

OPLS-DA analysis was performed on the MG and MHG to find biomarkers for MCS treatment of type 2 diabetes with NAFLD. The OPLS-DA model is of good quality, and the model evaluation indexes in positive ion mode are R2Y = 0.95, Q2 = 0.83, and the model evaluation indexes in negative ion mode are R2Y = 0.91, Q2 = 0.85. In the OPLS-DA score chart (Fig. 7a, b), the MG and MHG can be clearly divided into two parts, indicating that the difference between the groups is much larger than the difference between the groups. In S-plot (Fig. 7c, d), the points at both ends of the S-type are potential biomarkers, and the VIP > 1.0 and *p*-value< 0.05 between DG, MG and MHG are used as the criterion for another biomarker. Finally, 16 endogenous metabolites were identified as potential biomarkers (Table 3). Metabolic pathways affected by the biomarkers can be obtained by MetPA (http://metpa.metabolomics.ca/) analysis, including Taurine and hypotaurine metabolism, Pantothenate and CoA biosynthesis, Alanine, aspartate and glutamate metabolism, Riboflavin metabolism, Arginine biosynthesis, Citrate cycle (TCA cycle), Glyoxylate and dicarboxylate metabolism, Tryptophan metabolism, Primary bile acid biosynthesis, Fatty acid biosynthesis and Purine metabolism (Fig. 8a). Searching these metabolic pathways and biomarkers in KEGG database and establishing the metabolic correlation network and heatmap of metabolites affected by MCS treatment (Fig. 8b, c).

**Table 3:** Identification results of potential biomarkers

Mode	RT	Measured mass	VIP	Formula	Error (ppm)	Identification	Trenda
ESI+	7.19	206.0438	4.78	C10H7NO4	1.0	Xanthurenic acid	up
	2.59	338.1134	3.07	C7H14N2O6S	3.0	5-L-Glutamyl-taurine	up
	2.30	105.0414	2.91	C10H18N4O6	-6.7	Argininosuccinic acid	down
	1.18	220.1181	2.86	C9H17NO5	0.9	Pantothenic acid	up
	1.93	162.0673	2.80	C12H22N2O6S	0.6	D-Pantothenoyl-L-cysteine	down
	0.51	191.0206	2.18	C6H8O7	-2.0	Citric acid	up
	3.08	164.0711	2.13	C9H9NO2	-3.0	3-Methyldioxyindole	up
	8.04	426.3567	1.77	C25H47NO4	2.6	Vaccenyl carnitine	up
	1.70	135.0641	1.70	C5H10O4	-8.1	2,3-Dihydroxyvaleric acid	down
ESI-	2.74	143.1067	6.68	C8H16O2	-7.7	Caprylic acid	up
	0.48	167.0201	3.11	C5H4N4O3	6.0	Uric acid	down
	6.37	377.1454	2.79	C17H20N4O6	0.8	Riboflavin	up
	3.30	173.0808	2.32	C8H14O4	-6.4	Suberic acid	down
	0.40	124.0067	2.29	C2H7NO3S	6.4	Taurine	up
	1.13	154.0505	1.53	C7H7NO3	2.6	3-Hydroxyanthranilic acid	up
	2.24	157.0882	1.28	C8H14O3	1.9	3-Oxooctanoic acid	down

Metabolite change trend in MHG compared with MG

## Discussion

Mangiferin is widely found in many edible and medicinal plants and has many pharmacological activities, such as antitussive, expectorant, antiasthmatic, central depression, anti-diabetic, antioxidant, anti-inflammatory, bacteriostatic, anti-viral, anti-tumor, choleretic and immunomodulatory, so it has attracted the attention of researchers [14, 19]. Especially it has a good improvement effect on metabolic diseases such as diabetes, non-alcoholic fatty liver and hyperuricemia [27]. It has been reported that mangiferin under hypoxic conditions can promote the absorption of glucose by cells and improve insulin resistance and damage in fat cells [28]. It can significantly reduce blood glucose levels, increase glucose tolerance, increase serum insulin levels, and promote islet regeneration and β cell proliferation, and inhibit β cell apoptosis [29, 30]. In addition, mangiferin can reduce insulin resistance by regulating the redistribution of sarcolemma and intracellular fatty acid transfer enzymes in skeletal muscle [31]. It still can inhibit liver diacylglycerol acyltransferase gene expression, reduce liver quality and liver TG and TC levels, and inhibit excessive accumulation of lipid in the liver [32]. Although mangiferin has many pharmacological effects, due to its low solubility, it cannot be completely dissolved in the aqueous phase and the oil phase, has poor oral absorption, and has low bioavailability, which limits further clinical development and application [33]. At present, the modification of mangiferin derivatives and their metabolic active products may be an important direction for in-depth research and clinical application for it [34]. Mangiferin calcium salt (MCS) is a derivative of mangiferin, which may be an effective way to solving the above problems.

Therefore, we detected the blood drug concentration of MCS and mangiferin in single and multiple doses, and calculated their pharmacokinetic parameters in different time. Compared with mangiferin, the Tmax of MCS was advanced, and the AUC, Cmax of MCS increased significantly indicating that the degree of oral absorption of MCS was improved.

Shorter peak time showed that the rate of absorption of MCS was faster than the monomer of mangiferin. Moreover MCS has higher bioavailability than mangiferin. Compared the pharmacokinetic parameters between single and multiple dose oral administration of MCS, MRT and T1/2 had no significant change, which indicated that the absorption of MCS in rats is basically constant, and it will not change with continuous administration. These results showed that compared to mangiferin, MCS had a faster absorption rate, better absorption degree and its absorption was more constant.

IR plays a very key role in the pathogenesis of type 2 diabetes and NAFLD [35]. IR causes the body to produce compensation and secrete more insulin due to the body’s decreased glucose regulation function. This result leads to the hydrolysis of triglycerides in the body and the increase of plasma fatty acid content, which ultimately promotes the increase of blood sugar and is excreted from the kidney [36, 37]. At the same time, IR prevents insulin from efficiently inhibiting lipase activity. The increase in this enzyme activity will cause a large amount of fat to be broken down and enter the liver through the hepatic portal vein, causing simple fatty liver, which is related to oxidative stress Lipid peroxidation and the further action of inflammatory factors will lead to increased triglyceride content and destroy liver function [38–40]. Our previous research results show that MCS can significantly reduce fasting blood glucose and fasting insulin levels in rats with type 2 diabetes and NAFLD, reduce serum lipid levels, improve liver function, repair liver damage, and significantly increase the antioxidant capacity of model rats Ability to reduce oxidative stress and lipid peroxidation damage in model rats. It reveals that MCS has a certain therapeutic effect on type 2 diabetes and NAFLD. Moreover, 16 potential biomarkers related to type 2 diabetes and NAFLD were changed in the urine of MCS treated rats in our metabolomic study.

Among these metabolites, D-Pantothenoyl-L-cysteine is involved in the biosynthetic pathway of pantothenic acid and CoA, and is a synthetic precursor of Pantothenic acid that is a water-soluble vitamin required for life support. It is involved in the synthesis of acetyl-CoA and plays an important role in the metabolism of protein, fat, and sugar in the body [41]. Riboflavin is a prosthetic group of flavinases in the electron transfer process of the respiratory chain, which has anti-lipid peroxidation effect. As an important oxidoreductase in the body, flavinase participates in sugar oxidation metabolism and promotes the conversion of pyruvate to acetyl-CoA Process, thereby improving energy supply [42]. The average content of riboflavin in the urine of type 2 diabetes patients is generally lower than that of the normal population [43]. Caprylic acid, suberic acid and 3-oxooctanoic acid are important unsaturated fatty acids in the body. They regulate metabolism and cell signal transduction in the body, participate in the synthesis, decomposition and metabolism of fatty acids, and are converted into acetyl-CoA through beta oxidation into the citric acid cycle [44, 45]. Vaccenyl carnitine is a long-chain acyl fatty acid derivative of carnitine. Mitochondrial carnitine palmitoyl transferase II deficiency patients accumulate long-chain acyl fatty acid derivatives in the cytoplasm and serum [46]. It is a normal recessive disease of fatty acid metabolism. Abnormal oxidation of mitochondrial fatty acids can lead to hypoglycemia, liver dysfunction, myopathy, cardiomyopathy and encephalopathy [47, 48]. Argininosuccinic acid is a metabolite in the main biochemical pathway of lysine. It is an intermediate for the metabolism of lysine and sucralose. Studies in rats have shown that the level of argininosuccinic acid increases in pre-diabetes, so aminoadipate can be used as a predictive biomarker for the development of diabetes [49].

Xanthurenic acid, 3-Methyldioxyindole, 3-Hydroxyanthranilic acid are metabolite of tryptophan metabolism. Tryptophan and its metabolites play an important role in various physiological processes in the body, which mainly affect the immune system and nervous system. It is closely related to various diseases such as autoimmune diseases, abnormal liver function, CNS diseases and cancer [50]. 5-L-Glutamyl taurine is an intermediate of taurine metabolism. Taurine has many biological functions, such as cell membrane stabilizers and ion transmission accelerators, which can affect body fat metabolism, reduce inflammation and oxidative stress. Uric acid is a product of purine metabolism [51]. Abnormal purine metabolism can cause uric acid accumulation in the body, leading to gout, chronic kidney disease, diabetes, hyperlipidemia, hypertension and other diseases [52].

These metabolites are closely related to the occurrence and development of type 2 diabetes and NAFLD. In this study, MCS can exert its therapeutic effect by regulating the above metabolites.

## Conclusions

In summary, our results showed that the pharmacokinetic profiles of MCS were better than mangiferin. Also MCS had a good therapeutic effect on type 2 diabetes with NAFLD rats by regulating glycolipid metabolism. The metabolomics could provide effective information for metabolic changes in model rats after administration of MCS in urine. However the animal models do not fully reflect human NAFLD, and there are still some debates about the occurrence of NAFLD in T2DM. Our results might help to provide useful evidence for mechanism and clinical applications of MCS acting on type 2 diabetes and NAFLD.

## Data Availability

The datasets used and/or analyzed during the current study are available from the corresponding author on reasonable request.

## Abbreviations

MCS: Mangiferin calcium salt
NAFLD: Non-alcoholic fatty liver
IR: Insulin resistance
MGN: Mangiferin
HPLC: High performance liquid chromatography
STZ: Streptozotocin
BG: Blank control group
MG: Model control group
MHG: MCS High-dose group
MMG: Medium dose group
MLG: Low-dose group
FBG: Fasting blood glucose
FINS: Fasting insulin
TG: Triglyceride
TC: Total cholesterol
AST: Aspartate aminotransferase
ALT: alanine aminotransferase
GGT: gamma-glutamyl transpeptadase
H&E: Hematoxylin and eosin
UPLC-Q-TOF-MS: Ultra-performance liquid chromatography coupled with quadrupole time-of-flight mass spectrometry
QC: Quality control
PCA: Principal component analysis
OPLS-DA: Orthogonal projection to latent structures squares-discriminant analysis
